# Cell autonomous functions of CD47 in regulating cellular plasticity and metabolic plasticity

**DOI:** 10.1038/s41418-024-01347-w

**Published:** 2024-07-23

**Authors:** Ruhi Polara, Raja Ganesan, Stuart M. Pitson, Nirmal Robinson

**Affiliations:** 1 Centre for Cancer Biology, University of South Australia and SA Pathology, Adelaide, SA Australia; 2 Adelaide Medical School, Faculty of Health and Medical Sciences, The University of Adelaide, Adelaide, SA Australia; 3 School of Biological Sciences, The University of Adelaide, Adelaide, SA Australia; 4 Present Address: Institute for Molecular Immunology, CECAD Research Center, University Hospital Cologne, Cologne, Germany

**Keywords:** Cell biology, Biochemistry

## Abstract

CD47 is a ubiquitously expressed cell surface receptor, which is widely known for preventing macrophage-mediated phagocytosis by interacting with signal regulatory protein α (SIRPα) on the surface of macrophages. In addition to its role in phagocytosis, emerging studies have reported numerous noncanonical functions of CD47 that include regulation of various cellular processes such as proliferation, migration, apoptosis, differentiation, stress responses, and metabolism. Despite lacking an extensive cytoplasmic signaling domain, CD47 binds to several cytoplasmic proteins, particularly upon engaging with its secreted matricellular ligand, thrombospondin 1. Indeed, the regulatory functions of CD47 are greatly influenced by its interacting partners. These interactions are often cell- and context-specific, adding a further level of complexity. This review addresses the downstream cell-intrinsic signaling pathways regulated by CD47 in various cell types and environments. Some of the key pathways modulated by this receptor include the PI3K/AKT, MAPK/ERK, and nitric oxide signaling pathways, as well as those implicated in glucose, lipid, and mitochondrial metabolism. These pathways play vital roles in maintaining tissue homeostasis, highlighting the importance of understanding the phagocytosis-independent functions of CD47. Given that CD47 expression is dysregulated in a variety of cancers, improving our understanding of the cell-intrinsic signals regulated by this molecule will help advance the development of CD47-targeted therapies.

## Facts

- CD47 is a ubiquitously expressed cell surface receptor, widely known for its role in preventing phagocytosis through its interaction with SIRPα.
- CD47 also influences cellular behaviors beyond its “don’t eat me” signal function, including cellular and metabolic plasticity.
- Through its cytoplasmic tail, CD47 regulates cell-intrinsic functions.
- Depending on the cellular context and the ligand it binds to, CD47 modulates cellular responses to stress, cell-motility, migration, cell death and cell proliferation.
- Furthermore, it regulates cellular metabolism, including glycolysis, mitochondrial, fatty acid and nucleotide metabolism.

## Outstanding Questions

- What factors determine cell-type-specific function of CD47?
- How does CD47 regulate the cell-autonomous functions upon binding to a ligand?
- Is there crosstalk between CD47’s canonical and noncanonical functions?
- How does targeting CD47 affect its cell-autonomous functions?
- Can CD47 serve as a target for other autoinflammatory diseases?

## Introduction

### Cluster of differentiation 47 (CD47) structure and isoforms

CD47 (also known as IAP, MER6, or OA3) is a cell surface, integrin-associated glycoprotein belonging to the immunoglobulin (Ig) superfamily [1]. Structurally, it is composed of a single, glycosylated, extracellular variable Ig domain, a presenilin domain comprising five transmembrane-spanning segments, and a short variably spliced C-terminal cytoplasmic tail that gives rise to four isoforms [2, 3]. Isoform 2 is the most abundant isoform of CD47, which is expressed primarily by hematopoietic, endothelial, and epithelial cells [4]. Isoforms 3 and 4 are expressed predominantly in neural tissue, while isoform 1 is mainly present in keratinocytes [4]. Besides the proposed roles of isoforms 3 and 4 in memory retention and isoform 2 in transducing signals between the extracellular matrix (ECM) and cytoskeleton of astrocytes, the functional significance of alternate CD47 RNA splicing is poorly understood [5].

### CD47 ligands and binding part

Initially recognized for associating with the Rhesus (Rh) antigen complex on red blood cells (RBCs), subsequent early studies revealed that CD47 engages with ανβ3 integrin in human placenta and granulocytes and functions as an overexpressed tumor antigen in ovarian cancer [6–10]. Affinity labeling and CD47-deficient mouse model studies further demonstrated that thrombospondin 1 (TSP1), a secreted ECM glycoprotein, acts as a *trans*-spanning ligand for CD47, while signal regulatory protein α (SIRPα) serves as its cognate receptor [11, 12]. Subsequently, CD47 has been shown to interact with integrins, including αIIbβ3, α2β1, ανβ3, α4β1, α6β1, and αмβ2, as well as with caveolin-1, VEGFR2 and NOX1 in a *cis* configuration across different cell types [13–22]. In some cases, CD47, TSP1, and specific integrins form complexes that regulate downstream signaling [13–15]. Furthermore, CD47 associates with several cytoplasmic downstream signaling molecules such as BNIP3, PLIC-1, ENO1, AKAP13 and G_*i*_ signaling proteins [23–27]. Collectively, the interactions between CD47 and its binding partners play pivotal roles in regulating cellular processes such as migration, proliferation, adhesion, and phagocytosis.

### The canonical and noncanonical roles of CD47

The canonical role of CD47 is to act as a ‘don’t eat me signal’ to inhibit phagocytosis by macrophages through its interaction with SIRPα, thereby protecting cells from immune clearance and maintaining tissue homeostasis. Thus, the CD47- SIRPα interaction is crucial for preventing autoimmune reactions, maintaining immune tolerance, and regulating immune responses. The mechanisms through which the interaction between CD47 and SIRPα enables cells to evade phagocytosis are well characterized and have been extensively reviewed elsewhere [28–31]. However, numerous SIRPα-independent functions of CD47 have been identified [32, 33]. This review primarily focuses on the noncanonical, cell-autonomous functions of CD47, which are independent of macrophage-mediated phagocytosis.

## CD47 regulates cellular plasticity

### Cell stress and survival

Various cellular stressors differentially regulate cell fate. CD47 has been shown to regulate several types of stress responses, including response to radiation and oxidative stress [20, 22, 34, 35].

#### Autophagy-mediated response

**Fig. 1:**
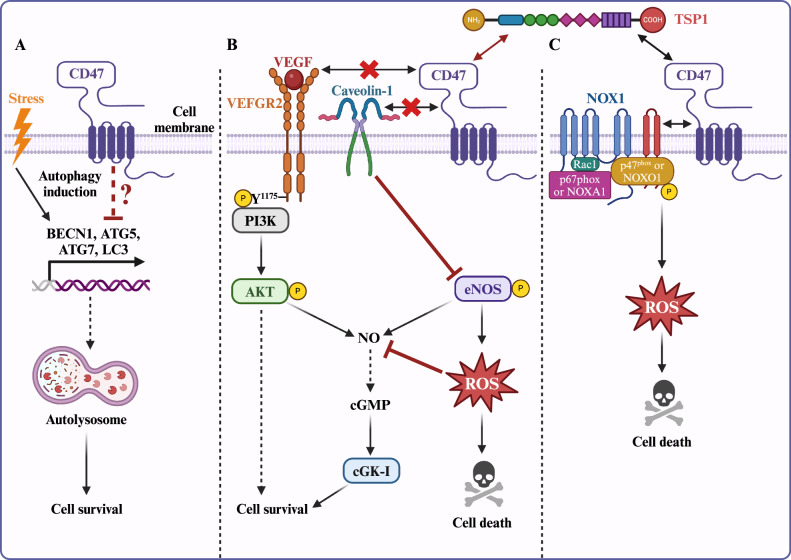
CD47 regulates cellular stress response pathways. **A** Under cellular stress, depleting CD47 triggers activation of gene expression for beclin-1 and autophagy-related genes ATG5 and ATG7. The upregulation of beclin-1, ATG5, and ATG7 enhances autophagic flux by increasing the expression of LC3, which is essential for forming the autophagosome membrane. Consequently, unwanted cellular components are targeted and degraded within autophagosomes, ultimately promoting cell survival. **B** TSP1 binding with CD47 disrupts constitutive association between CD47 and VEGFR2 on endothelial cells, effectively blocking VEGFR2 induced PI3K/AKT-mediated activation of eNOS and subsequent induction of NO/cGMP signaling and other signaling pathways in favor of cell survival. Additionally, TSP1 disrupts CD47 interaction with caveolin-1 on endothelial cells to enhance reactive oxygen species (ROS) production via eNOS which can also contribute to NO/cGMP signaling to enhance cell survival. **C** TSP1-CD47 engagement activates NOX1 through p47^phox^ phosphorylation, resulting in ROS production and cell death. This figure was created using BioRender.com.

Exposure to ionizing radiation induces acute DNA damage, which if left unrepaired, can lead to cell death. In these irradiated cells, autophagy acts as a protective mechanism by removing damaged organelles, proteins, and cellular components to restore homeostasis. CD47 exacerbates the response to radiation-induced stress by inhibiting autophagy. For instance, experiments in the Jurkat immortalized CD4^+^ T cell line have shown that CD47 depletion promotes cell survival and proliferation following exposure to ionizing radiation by inducing autophagy [36]. CD47-depleted cells are characterized by increased autophagosome formation and transcription of autophagy-related genes such as *BECN1, ATG5*, and *ATG7* [36]. Moreover, silencing *ATG5* and *ATG7* sensitizes CD47-deficient Jurkat cells to ionizing radiation, confirming the key role of autophagy in regulating CD47-mediated radiosensitivity (Fig. 1A). On the contrary, radiation-resistant breast cancer cells and irradiated breast tumors are found to rely on high CD47 expression for their survival [37]. However, the role of autophagy has not been investigated. Further extensive studies are required to understand the cell type specific role of CD47 in regulating autophagy.

#### Response to oxidative stress

Recent studies have also demonstrated an important role of CD47 in regulating oxidative stress responses triggered by ionizing radiation and ischemic stress. For instance, CD47-deficient Jurkat cells are better able to tolerate oxidative stress than their wild-type counterparts following exposure to ionizing radiation. This is evidenced by a significant increase in the glutathione redox couple potential and the sustained production of key components of the glutathione pathway, including cystathionine, glutamate, γ-glutamylcysteine, and 5-oxoproline [38]. Additionally, the level of S-lactoylglutathione, which is critical for metabolizing methylglyoxal (a highly reactive dicarbonyl compound), is higher in CD47-deficient than wild-type Jurkat cells post-irradiation [38]. Similarly, knocking out CD47 in mouse lung tissues increases the ratio of reduced to oxidized glutathione, suggesting that CD47 depletion enables cells to more effectively respond to oxidative stress induced by ionizing radiation [35].

In endothelial cells, CD47 establishes a constitutive interaction with VEGF receptor 2 (VEGFR2) to regulate PI3K/AKT-mediated activation of eNOS and subsequent NO production, contributing to the induction of NO/cGMP signaling [21, 39]. However, TSP1 binding to CD47 disrupts its interaction with VEGFR2, and consequently suppresses the NO-mediated cellular stress response [21] (Fig. 1B). Furthermore, during ischemic stress or ischemia-reperfusion injury, TSP1-CD47 interaction inhibits NO/cGMP signaling pathway which reduces vascular remodeling, diminishes tissue perfusion, and ultimately limit overall tissue survival [22, 40–45]. In contrast, under hypoxic conditions, TSP1 promotes endothelial NO synthase (eNOS) activity by disrupting the constitutive association between CD47 and caveolin-1, which paradoxically leads to increased superoxide production instead of NO [20]. This heightened oxidative stress contributes to vasoconstriction and a subsequent reduction in blood flow. In addition, TSP1 has been shown to suppress pro-survival responses in vascular smooth muscle cells (VSMCs) via its effects on the canonical NO/cGMP pathway [41, 46]. In addition to the TSP1-mediated suppression of pro-survival responses in VSMCs via NO/cGMP pathway, CD47 and TSP1 engagement can increase oxidative stress in these cells through the phosphorylation of p47^phox^, a NADPH oxidase (NOX) subunit, by phospholipase C and protein kinase C. In this scenario, subsequent NOX1 activation impairs arterial vasodilation and exacerbates oxidative stress [22] (Fig. 1C). Radioprotection in normal tissues in the absence of CD47 or TSP1 may also be partially attributed to the cytoprotective effects of NO signaling [47]. Furthermore, TSP1-CD47 association induces NO-mediated cell death of RBCs, in part by promoting calcium influx [48]. Interestingly, CD47-induced radiosensitivity is specific to healthy tissues, as inhibiting CD47 in mice bearing melanoma or squamous lung tumors prior to irradiation significantly reduces tumor growth [49]. However, whether this effect is dependent on NO or NOX signaling remains to be investigated.

Although loss of CD47 has been shown to enhance anti-oxidative response to ionizing radiation, TSP1 engagement with CD47 promotes oxidative stress via the activation of NO/cGMP and NOX signaling in a context-dependent manner. Therefore, further investigation is required to establish the precise role of CD47 in maintaining redox homeostasis.

#### Regulation of cell death

**Fig. 2:**
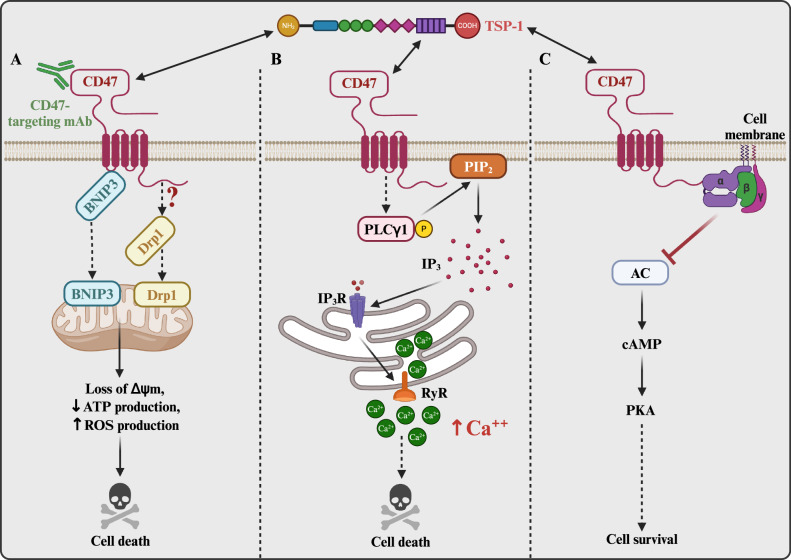
CD47-mediated regulation of cell death. **A** CD47 binds to intracellular BNIP3, an interaction disrupted following CD47 ligation with TSP1 or anti-CD47 targeting antibody. Following dissociation, BNIP3 translocates to the mitochondria, inducing depolarization, ultimately triggering cell death. Concurrently, TSP1-CD47 interaction prompts DRP1 translocation to the mitochondria, inducing further mitochondrial destabilization. **B** TSP1 binds to CD47 leading to sustained activation of PLCγ1, which then cleaves phosphatidylinositol 4,5-bisphosphate (PIP_2_) into inositol 1,4,5-trisphosphate (IP_3_). IP_3_ binds to its receptors (IP_3_R) on the endoplasmic reticulum (ER), prompting the release of Ca^2+^. This release activates the ER ryanodine receptors (RyR), further triggering calcium release from the ER into the cytoplasm. Calcium overload in the cell causes mitochondrial damage, ultimately leading to cell death. **C** CD47 and heterotrimeric G_*i*_ protein (α, β, γ) interaction activates G_*i*_ signaling, suppressing adenyl cyclase (AC), reducing cAMP levels, and diminishing PKA activity, which results in cell death. This figure was created using BioRender.com.

CD47 regulates cell death across various cell types, including B-cell chronic lymphocytic leukemia (B-CLL), T cell acute lymphoblastic leukemia (T-ALL), and breast cancer cells, as well as certain healthy cell lineages [50–54]. In leukemic B cells, the binding of CD47 to an immobilized anti-CD47 antibody or TSP1, orchestrates the translocation of dynamin-related protein 1 (DRP1) from the cytosol to the mitochondria [55]. The subsequent activation of DRP1 in turn disrupts the mitochondrial electron transport chain, triggering loss of mitochondrial membrane potential (∆Ψm), reactive oxygen species (ROS) production, exposure of phosphatidylserine (PE), and eventually, caspase-activation-independent cell death (Fig. 2A).

Like DRP1, BCL-2/adenovirus E1B 19 kDa protein-interacting protein 3 (BNIP3) has emerged as a critical regulator of CD47-mediated cell death [23]. BNIP3 specifically interacts with the cytoplasmic region of the transmembrane domain of CD47. Stimulation of CD47 with 4N1K, a TSP1-derived peptide which comprises the CD47 binding site, causes BNIP3 to dissociate from CD47 and translocate to the mitochondrion, where it depolarizes the mitochondrial membrane and triggers cell death (Fig. 2A). Overexpression of the anti-apoptosis protein BCL-2 in the presence of an anti-CD47 antibody antagonizes this process, emphasizing the interplay between BNIP3 and BCL-2 in orchestrating cell death downstream of CD47-TSP1 engagement [23].

In addition to TSP1 and 4N1K, the PKHB1 peptide (a more stable variant of the 4N1K peptide) elicits cell death in B-CLL cells, while sparing normal B lymphocytes [56]. PKHB1 treatment induces sustained activation of phospholipase C gamma-1 (PLCγ1) [56], which catalyzes inositol 1,4,5-trisphosphate (IP_3_) synthesis. Binding of IP_3_ to its receptor (IP_3_R) in the endoplasmic reticulum triggers store-operated calcium release, leading to actin depolymerization, mitochondrial damage, and subsequent cell death [56] (Fig. 2B). Interestingly, this process occurs independently of DRP1 activation, suggesting that PKHB1/4N1K, anti-CD47 antibodies, and full-length TSP1 each elicit unique forms of caspase-independent cell death in B-CLL cells.

CD47 also sensitizes Jurkat cells to radiation and topoisomerase inhibitors by upregulating the expression of Schlafen family member 11 (SLFN11), a key molecule that stimulates irreversible replication block and cell death under replication stress [57]. SLFN11 expression is inhibited by the binding of CD47 to TSP1, emphasizing a critical role of TSP1 in regulating CD47-mediated cell death [57]. At present, the exact mechanism of the CD47-mediated induction of SLFN11 is unclear. Furthermore, in breast cancer cells, CD47 participates in G_*i*_-mediated caspase-independent cell death [52]. The binding of CD47 to 4N1K or anti-CD47 antibody triggers heterotrimeric G_*i*_ signaling, resulting in reduced cAMP levels, consequent decrease in protein kinase A (PKA) activity, and ultimately cell death (Fig. 2C). This response is counteracted by the activation of PI3K/AKT signaling following EGFR stimulation, demonstrating a key role of this pathway in preventing CD47-mediated cell death [52].

Most studies have reported a pro-apoptotic role of TSP1-CD47 interaction, but it has also been shown to promote survival of cutaneous T lymphoma cells in vitro and enhance tumor growth in vivo [58]. Although the detailed mechanism underlying this paradoxical, anti-apoptotic role of CD47-TSP1 remains to be determined, increased ERK1/2 and AKT phosphorylation, coupled with the elevated Survivin expression, have been observed upon CD47-TSP1 engagement, suggesting the potential involvement of this signaling in governing cell survival downstream of CD47 [58]. Moreover, CD47-TSP1 interaction protects thyroid carcinoma cells from camptothecin- or doxorubicin-induced caspase-mediated apoptosis [59]. Similarly, the TSP1-derived 4N1K peptide plays a protective role by counteracting ceramide-induced caspase-3-dependent apoptosis primarily through cAMP/PKA signaling in thyroid cells [60]. It must be noted, however, that several CD47-independent activities of the 4N1K peptide have been documented [61, 62]. Therefore, results obtained using 4N1K, which have not been validated using native TSP1, inhibitory anti-CD47 antibodies, or CD47-*null* cells, should be interpreted with caution.

Taken together, these findings indicate that CD47 functions are highly dependent on its interacting partner. This could likely result in cell type-specific roles of CD47 in regulating cell death or survival which could be further influenced by the cellular environment.

### Cell adhesion, motility, and migration

**Fig. 3:**
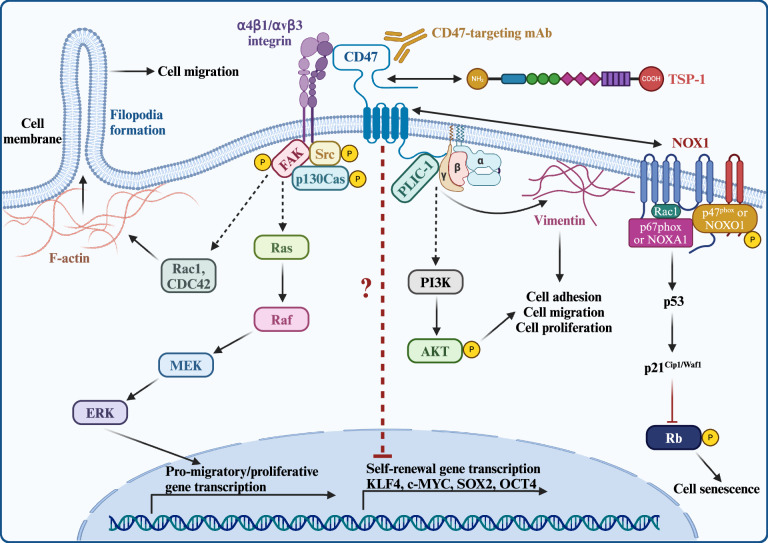
Mechanisms underlying CD47-regulated cellular plasticity. From left to right: CD47 associates with integrins α4β1 or ανβ3, inducing the assembly and activation of the focal adhesion complex (FAC). The FAC, composed of Src, focal adhesion kinase (FAK), and p130Cas, in turn stimulates Rac1 and CDC42 and/or MEK/ERK signaling, promoting cell migration and proliferation via increased F-actin expression and filopodia formation or the induction of gene expression, respectively. Upon CD47 activation by TSP1, CD47 associates with intracellular PLIC-1, which tethers vimentin filaments to the cell membrane, facilitating integrin-ανβ3-mediated cell spreading. PLIC-1 also forms a complex with the Gβγ dimer, stimulating PI3K/AKT signaling to promote cell motility and proliferation. TSP1-CD47 signaling further regulates cell self-renewal by downregulating SOX2, OCT4, KLF4, and c-MYC expression. TSP1 binding to CD47 also activates NOX1, inducing reactive oxygen species (ROS) generation and initiating a p53-mediated DNA damage response. This leads to p21^Cip1/Waf1^ upregulation and the subsequent hypophosphorylation of retinoblastoma protein (Rb), ultimately resulting in cell senescence. This figure was created using BioRender.com.

Impaired cell adhesion, motility, and migration underlies the pathophysiology of many metastatic cancers and immunodeficiency disorders. CD47 has emerged as a key player to promote these processes across diverse cell types, including various cancer cells. CD47-TSP1 interaction has been shown to facilitate sickle RBC adhesion in an integrin-α4β1-dependent manner [63]. Mechanistically, CD47 and TSP1 ligation triggers G_*i*_- and PKA-dependent phosphorylation of the α4 integrin cytoplasmic domain and promotes Src-dependent sickle RBC adhesion to VCAM-1, fibronectin and immobilized TSP1 [63]. Experiments using 4N1K-induced CD47 activation have shown that the chemotaxis of smooth muscle cells (SMCs) towards collagen-I does not occur in the absence of CD47 [64]. In the presence of CD47, however, stimulation with 4N1K induces the G_*i*_-mediated inhibition of ERK via integrin α2β1, which lowers cAMP levels to promote SMC chemotaxis [64]. The functional interplay between integrin α2β1 and CD47 further extends to intestinal epithelial cells, in which this interaction facilitates cell migration by enhancing Gα_*i3*_-induced COX-2 expression [65]. Moreover, the association between CD47, TSP1, and integrin ανβ3 promotes the vitronectin-associated spread of melanoma cells by activating focal adhesion kinase (FAK), paxillin, and G_*i*_ signaling [66]. The direct binding of CD47 to G_*i*_ proteins, coupled with its interaction with integrin ανβ3 in melanoma cells, highlights a potential mechanism by which CD47 regulates G_*i*_ signaling to promote cell migration [26] (Fig. 3). Furthermore, CD47 associates with protein linking IAP with cytoskeleton 1 (PLIC-1), which is known to modulate G_*i*_-mediated cell migration [24, 67]. PLIC-1, which tethers to CD47 via its cytoplasmic tail and anchors vimentin filaments to the cell membrane, has been shown to promote the integrin-ανβ3-mediated spread of ovarian cancer cells [67] (Fig. 3). Intriguingly, Jurkat cells exhibit increased rates of migration in response to anti-CD47 antibody treatment, in a process which is sustained by PLIC-1 overexpression [67]. Interestingly, this effect is independent of integrin activation, indicating that integrins may not be required for the CD47-induced migration of certain cell types.

Inhibition of CD47 using anti-CD47 antibodies has been shown to impede both trans-endothelial and trans-epithelial migration of neutrophils, which is partially reversed by inhibiting PI3K [68–70]. Subsequent investigations have revealed that CD47 activates PI3K/AKT/mTOR signaling to promote the migration of endometrial carcinoma cells [71]. While several studies have implicated PI3K/AKT and G_*i*_ signaling pathways in the regulation of cell migration downstream of CD47, the CD47-induced motility (evidenced by increased lamellipodia formation) and migration of Madin-Darby canine kidney (MDCK) cells are not reliant on PI3K/AKT or G_*i*_ activation. In these cells, CD47 instead stimulates Src and MEK/MAPK signaling [72]. Similarly, CD47 promotes MAPK/ERK activation in adamantinomatous craniopharyngioma cells to support epithelial-to-mesenchymal transition (EMT)-induced cell migration [73]. This mechanism is also employed by colorectal cancer cells, whereby CD47 overexpression significantly increases ERK activity and promotes cell migration [25]. Collectively, these findings reveal the complex interactions between CD47 the various signaling pathways governing cell behavior.

In accordance with MDCK cells, recent investigations in intestinal epithelial cells have revealed that CD47 interacts directly with integrin β1 to promote the phosphorylation of Src^Y416^, FAK^Y397^, FAK^Y861^, and p130Cas^Y410^, facilitating focal adhesion complex (FAC) formation and increasing cell motility [74]. Although the precise mechanism via which CD47 activates Src remains unclear, it is worth noting that upon stimulation, integrin β1 directly binds and activates FAK to promote FAC assembly [75, 76]. This, in turn, activates various signaling pathways, including the MEK/MAPK pathway, ultimately facilitating cell migration [75] (Fig. 2). In platelets, the CD47- and integrin-αIIbβ3-induced cell spreading mediated by FAK and c-Src is triggered by TSP1, highlighting the key role of TSP1 in CD47-dependent FAC formation and cell migration [13]. Notably, this process is countered by the inhibition of G_*i*_ signaling, suggesting that the G_*i*_ pathway modulates platelet motility via FAK/c-Src activation downstream of CD47 [13]. The mechanism linking G_*i*_ signaling to Src stimulation following CD47 activation requires further elucidation.

The cytoplasmic tail of CD47 engages with AKAP13, a RhoA-specific guanine nucleotide exchange factor, to activate RhoA and increases the growth and metastasis of T cell lymphoma in vivo [27]. Interestingly, the expression of a chimeric protein composed of the cytoplasmic tail of CD47 and the extracellular domain of EGFR in T lymphoma cells lacking endogenous CD47 significantly increases their RhoA activity [27]. Thus, the cytosolic domain of CD47 can autonomously increase RhoA activity through modes of activation that bypass the need for ligand engagement. In addition to RhoA, CD47 influences other Rho-family GTPases, which are pivotal in lamellipodia and filopodia formation, to regulate cell motility. For instance, CD47 promotes neurite and filopodia formation by activating Rac1 and CDC42 in neurons and neuroblastoma cells [77] (Fig. 3). Similarly, CD47 enhances migration of non-small cell lung cancer cells by inducing the expression of CDC42 [78].

Collectively, these findings suggest that CD47 intricately governs cell adhesion, motility, and migration by regulating diverse signaling pathways. The complexity of these signaling networks highlights the need for comprehensive research to explore the multifaceted role and potential therapeutic applications of CD47 modulation in conditions characterized by the impairment of these cellular processes.

### Cell proliferation

Besides regulating cell adhesion and migration, CD47 serves as a central regulator of cell proliferation. CD47 has been shown to promote proliferation of colorectal cancer and adamantinomatous craniopharyngioma cells [25, 73] (Fig. 3). Moreover, 4N1K-induced stimulation of CD47 signaling promotes proliferation of astrocytoma cells, which is attenuated by CD47 blockade [79]. Mechanistically, CD47 activation induces cell proliferation by engaging with PLIC-1 and the Gβγ dimer, triggering PI3K/AKT signaling [79]. CD47 stimulation by 4N1K also promotes proliferation of glioblastoma cells by increasing the expression of ubiquitin-like containing PHD and RING Finger 1 (UHRF1) proteins while reducing the expression of tumor suppressor p16^INK4A^ [80, 81]. Intriguingly, normal astrocytes are unaffected by the presence of 4N1K, suggesting that CD47 selectively enhances the proliferation of tumor cells [79, 80].

CD47 also mediates thrombin-induced nuclear export of p21 cyclin-dependent kinase-interacting protein 1 (p21^Cip1/Waf1^, also known as CDKN1A) and its subsequent cytoplasmic degradation, facilitating aortic smooth muscle cell proliferation [82]. Furthermore, CD47 signaling induces proliferation in Epstein-Barr virus (EBV)-transformed B cells [83]. In accordance, inhibition of CD47 signaling with a blocking anti-CD47 antibody suppresses the activation of ERK1/2 and PI3K/Akt-mTOR signaling pathways, while inducing the ROS-mediated activation of the p38 MAPK/JNK pathway [83]. This cascade results in the upregulation of TAp73 expression, induction of endoplasmic reticulum stress, G_1_ cell-cycle arrest, and ultimately inhibition of cell proliferation. Notably, TSP1 treatment, recapitulates G_1_ cell-cycle arrest induced by CD47 inhibition, indicating that TSP1 may differentially regulate CD47-mediated cell proliferation across various cell types [83].

### Self-renewal and differentiation

The regulation of stem cell self-renewal implicates numerous transcription factors, including octamer-binding transcription factor 4 (OCT4), sex-determining region Y-box 2 (SOX2), Krüppel-like factor 4 (KLF4), and the cellular homolog of the v-myc avian myelocytomatosis viral oncogene homolog (c-MYC) [84, 85]. The forced expression of these transcription factors has been demonstrated to induce self-renewal in both human and mouse somatic cells [86]. Recent studies have shown that the TSP1-CD47 interaction inhibits self-renewal of intestinal epithelial cells [87] and lung endothelial cells [88] by downregulating OCT4, KLF4, SOX2, and c-MYC (Fig. 3). Similarly, TSP1-CD47 engagement inhibits self-renewal of renal tubular epithelial cells by reducing c-MYC and SOX2 expression [89]. Remarkably, CD47-deficient cells efficiently form embryoid-body-like clusters containing pluripotent cells which exhibit high rates of proliferation and differentiation into cell types comprising all three embryonic germ layers [88].

Contrary to its role in untransformed healthy cells, CD47 supports self-renewal of cancer stem cells (CSCs) in breast cancer and hepatocellular carcinoma [90, 91]. Blocking CD47 leads to downregulation of KLF4, and EGFR expression, potentially mediated by the upregulation of miR-7. This, in turn, inhibits asymmetric division and promotes differentiation of breast CSCs [91]. Meanwhile, CD47 regulates tumor initiation and stemness of hepatocellular carcinoma stem cells by triggering the secretion of cathepsin S, which stimulates NF-κB and protease-activated receptor-2 (PAR-2) signaling [90]. This activation amplifies cathepsin S release, establishing a positive feedback loop [90]. The precise mechanism via which CD47 modulates cathepsin S secretion in hepatocellular carcinoma and potentially other cell types remains to be elucidated.

### Cellular senescence

Cellular senescence, triggered by diverse endogenous and exogenous stresses such as telomere dysfunction, oncogene activation, and persistent DNA damage, is a critical process associated with tissue degeneration, cell exhaustion, and aging [92]. To date, CD47 has been shown to induce senescence in endothelial cells, colorectal cancer cells, and breast cancer cells [93, 94]. For instance, TSP1-CD47 engagement has been shown to promote the senescence of endothelial cells, which is associated with reduced β-galactosidase (SA-β-gal) activity and increased cell-cycle progression [93]. Conversely, knocking out CD47 attenuates endothelial cell senescence even in the presence of TSP1 [93]. At the molecular level, TSP1 activates NOX1-dependent generation of ROS, initiating p53-mediated DNA damage responses, which leads to upregulation of p21^Cip1/Waf1^ and a concurrent decrease in retinoblastoma protein (Rb) phosphorylation, ultimately resulting in cell senescence [95] (Fig. 3).

In colorectal and breast cancer cells, TSP1-CD47 interaction prevents senescence escape following chemotherapy treatment [94]. CD47 downregulation correlates with reduced p21^Cip1/Waf1^ and elevated Ki67 expression, suggesting that CD47 plays an important role in maintaining senescence in these cells. Notably, inactivation of p21^Cip1/Waf1^ upregulates c-MYC expression which can further influence CD47 levels to regulate senescence, demonstrating a reciprocal link between CD47, p21^Cip1/Waf1^, and c-MYC [94]. These findings indicate that targeting CD47 in combination with chemotherapy should be undertaken with caution as CD47 inhibition could potentially promote senescence escape and chemotherapy resistance, fostering a more aggressive tumor phenotype.

## CD47 regulates metabolic plasticity

### Mitochondrial metabolism

**Fig. 4:**
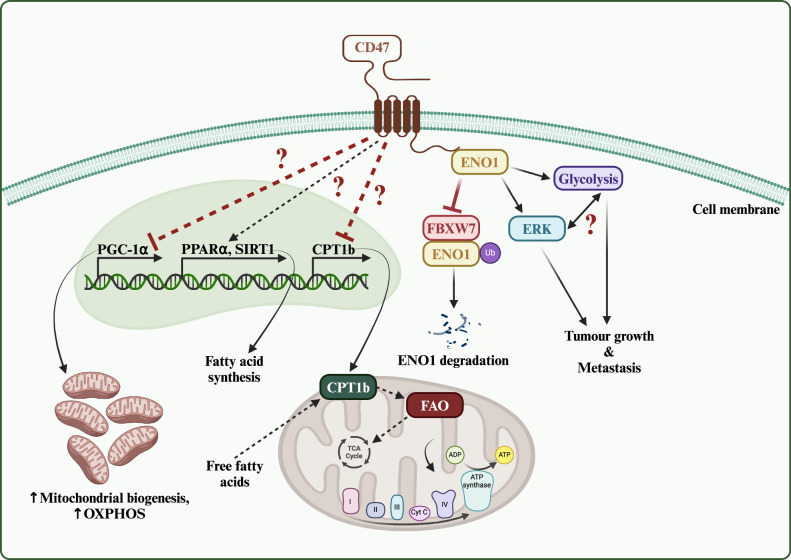
CD47 regulates metabolic remodeling. From left to right: Loss of CD47 induces the expression of PGC-1α, enhancing mitochondrial biogenesis and function, consequently increasing oxidative phosphorylation (OXPHOS). Additionally, CD47 dynamically modulates the expression of PPARα and SIRT1, regulating fatty acid synthesis in response to varying dietary conditions. Through the transcriptional downregulation of CPT1b, CD47 also suppresses fatty acid oxidation. Moreover, CD47 interacts with Enolase 1 (ENO1) to inhibit its FBXW7-mediated degradation. The CD47-ENO1 interaction activates ERK signaling and promotes glycolysis in favor of tumor growth and metastasis. This figure was created using BioRender.com.

Cells constantly undergo metabolic shifts to grow, function, and survive. This dynamic process is particularly evident in cancer cells, which rapidly adapt to challenging environments such as hypoxia, nutrient deprivation, and other cellular stressors [96]. Recently, CD47 has been implicated in the regulation of mitochondrial metabolism, which is crucial in the coordination of diverse cellular processes necessary for cellular adaptation [97]. In skeletal muscle and Jurkat cells, CD47 deficiency increases mitochondrial mass and elevates the expression of PGC-1α, a key transcriptional coactivator of mitochondrial biogenesis [35, 98]. This increase in mitochondrial biogenesis promotes mitochondrial respiration [35] (Fig. 4). In accordance, CD47 overexpression in colorectal cancer cells reduces oxygen consumption rate, which indicates a drop in the rate of mitochondrial respiration [25]. Interestingly, while the majority of tricarboxylic acid (TCA) cycle substrates and intermediates remain largely unaltered following CD47 depletion, citrate levels are significantly reduced. This is accompanied by elevated levels of acetylated peptides, suggesting that CD47 redirects citrate towards the synthesis of acetylated peptides, which are implicated in cell-cycle progression, cytoskeletal dynamics, chromatin remodeling, and membrane trafficking [99]. These findings suggest that in addition to its role in mitochondrial biogenesis via PGC-1α, CD47 could be regulating TCA metabolic flux.

Although CD47 depletion promotes mitochondrial respiration in Jurkat cells, basal oxygen consumption rates are similar between CD47-*null* and wild-type white adipocytes [35, 100]. By contrast, the mitochondria of brown adipocytes derived from CD47-deficient mice consume more oxygen during fatty acid oxidation than those derived from wild-type animals [100]. This suggests that CD47 differentially regulates mitochondrial function in various cell types, which may be altered in response to various stimuli, such as changes in nutrient availability or exposure to stressors. NO/cGMP signaling also regulates mitochondrial biogenesis and increased levels of cGMP have been detected in white adipocytes lacking CD47 [100]. Thus, NO/cGMP signaling may promote mitochondrial biogenesis downstream of CD47. Further investigation is required to validate these outcomes and fully understand the role of CD47 in mitochondrial metabolism.

### Glucose metabolism

In addition to regulating mitochondrial metabolism, CD47 modulates glucose uptake and glycolysis [25, 35]. Jurkat cells lacking CD47 increase their glucose uptake, which is evidenced by elevated GLUT1 expression and 2-NBDG assimilation [35]. Despite exhibiting higher glucose uptake, CD47-deficient cells have lower basal levels of glycolytic intermediates (e.g., glucose-6-phosphate and fructose 6-phosphate) in lung tissues of CD47-depleted mice than in those of wild-type animals, indicating a reduction in the downstream regulation of glycolytic flux [35]. Importantly, CD47-deficient cells exhibit more stable levels of several glycolytic, and TCA cycle metabolites (e.g., fructose 1,6-bisphosphate, pyruvate, malate, fumarate), as well as 5-methyltetrahydrofolate and pyrophosphate, which are implicated in DNA damage repair following irradiation [35]. This suggests that CD47 possibly regulates the glycolytic enzymes or redirects glucose through alternate metabolic pathways that lead to the synthesis of folate derivatives, which remains to be investigated. Meanwhile, CD47 overexpression increases the levels of glucose-6-phosphate, phosphoenolpyruvate (PEP), pyruvate, and lactate in colorectal cancer cells [25]. This is accompanied by the upregulation of ERK signaling and the subsequent increase in cell growth and metastasis. Mechanistically, CD47 competitively interacts with the glycolytic enzyme Enolase 1 (ENO1) to inhibit its binding to FBXW7, an E3 ubiquitin ligase, thereby preventing ENO1 degradation. Consequently, the stabilization of ENO1 promotes glycolysis and the activation of ERK signaling in favor of cell proliferation and metastasis [25] (Fig. 4). Whether increased glucose metabolism contributes to mitochondrial metabolism remains to be investigated. Furthermore, how changes in glycolytic flux regulates growth kinases such as ERK and regulate cell proliferation needs to be investigated mechanistically.

### Nucleotide metabolism

Nucleotide biosynthesis plays an important role in supporting the activation of DNA repair mechanisms following the generation of ionizing-radiation-induced double-stranded DNA breaks. Recent studies have unveiled the regulatory role of CD47 in nucleotide metabolism, especially in response to ionizing radiation exposure. CD47-depleted Jurkat cells subjected to ionizing radiation exhibit significantly elevated concentrations of 5′-monophosphate, a crucial intermediate in purine nucleotide biosynthesis [35]. Furthermore, loss of CD47 stabilizes levels of adenine and guanine nucleotides derived from inosine monophosphate [35]. Notably, CD47 deficiency also impacts pyrimidine nucleotide biosynthesis, as evidenced by reduced levels of uridine 5′-monophosphate and downstream metabolites in irradiated wild-type but not CD47-depleted cells [35]. Consistently, loss of CD47 enhances pyrimidine and purine biosynthesis in irradiated mouse lung tissue [38]. These data are consistent with decrease in glycolytic intermediates such as glucose-6-phosphate and hexose-6-phosphate despite increased glucose uptake, which suggests that glucose is possibly redirected to hexose shunt pathway resulting in increased nucleotide biosynthesis. Taken together, these findings demonstrate that CD47 deficiency protects nucleotide biosynthesis pathways and facilitates tissue recovery after radiation exposure.

### Fatty acid metabolism

Accumulating evidence suggests that CD47 is an important regulator of fatty acid metabolism. CD47 deficiency significantly increases lipid accumulation in the livers of mice that are fed a high-fat diet [101]. Mechanistically, the extent of liver fat deposition is associated with downregulation of peroxisome proliferator-activated receptor (PPARα) and Sirtuin 1 (SIRT1), two key regulators of lipid metabolism [101]. By contrast, feeding CD47-deficient mice a low-fat diet increases their PPARα and SIRT1 expression, implying that CD47 modulates the expression of these proteins in response to varying dietary conditions [101]. Moreover, the combination of CD47 deficiency and a high-fat diet stimulates the expression of uncoupling protein 1 (UCP1) and carnitine palmitoyltransferase 1b (CPT1b) in brown adipose tissue, which drives fatty acid oxidation [102]. Collectively, these findings indicate that CD47 regulates fatty acid metabolism via its effects on PPARα, SIRT1, UCP1, and CPT1b (Fig. 4). However, the underlying signaling pathways involved in mediating CD47-dependent regulation of these factors remain to be elucidated.

## Crosstalk between canonical and noncanonical functions of CD47

It is also important to note that crosstalk likely occurs between the canonical and noncanonical functions of CD47. The crosstalk between these pathways allows CD47 to coordinate complex cellular responses. For instance, given that CD47 associates with TSP1 and integrin αvβ3, it seems conceivable that CD47 may interact with integrin αvβ3 in the phagocytic clearance of apoptotic cells, where TSP1 may function as a bridging molecule [103]. It is thus possible that TSP1 interacts with apoptotic-cell-associated CD47 in this scenario. While CD47-SIRPα interaction prevents phagocytosis, the noncanonical pathways involving integrins and TSP1 can modulate immune cell migration and activation, fine-tuning the immune response. In cancer, CD47 not only inhibits phagocytosis via SIRPα but also affects tumor growth and metastasis through integrin signaling and modulation of angiogenesis. Moreover, the involvement of SIRPα in these processes underscores the broader regulatory implications of the CD47-SIRPα interaction beyond phagocytosis. CD47 promotes cell adhesion by interacting with SIRPα which has been elucidated using an extracellular SIRPα-human Ig fusion protein to promote the CD47-mediated adhesion of B-cell acute lymphoblastic leukemia cells by inducing PI3K activation [104]. Furthermore, during tissue injury and repair, CD47’s role in preventing phagocytosis ensures cell survival, while its interactions with integrins and TSP1 can influence cell migration and new tissue formation. Therefore, while developing approaches to therapeutically target CD47, its canonical and noncanonical functions must be considered.

## CD47 as a therapeutic target

CD47 is overexpressed in a variety of cancers. Cancer cells preferentially express CD47 as a ‘don’t eat me signal’, which protects them from macrophage-mediated phagocytosis. Thus, targeting the interaction between CD47 and its SIRPα receptor has emerged as a potential therapeutic strategy for cancer treatment [105, 106]. The feasibility of using anti-CD47 and -SIRPα blocking antibodies for the treatment of various cancers is currently being evaluated in phase I/II clinical trials [107–110]. Targeting CD47 to disrupt its interaction with SIRPα can enhance the immune system’s ability to destroy cancer cells and could be explored to prevent autoinflammatory diseases. Anti–neutrophil cytoplasmic antibody (ANCA)–associated vasculitis is an autoinflammatory disease in which ANCA triggers neutrophils to induce neutrophil extracellular traps (NET) which promotes vascular injury. NETs associated with vasculitis are thought to escape efferocytosis due to the expression of CD47 and CD47 blockade has been shown to mitigate ANCA-associated vasculitis [111]. Concurrently, modulating its noncanonical pathways can inhibit tumor growth and metastasis. Furthermore, leveraging CD47’s roles in cell survival and migration can improve tissue repair and regeneration strategies. To date, however, the ubiquitous expression of CD47 on healthy cells causes off-tumor toxicities in most patients [109, 110]. Off-tumor toxicity arises from the unintended impact on normal cells and physiological processes. CD47 is expressed on various cells such as RBCs and platelets and therapies targeting CD47 can lead to their phagocytosis, resulting in anemia and thrombocytopenia respectively. Furthermore, a recent study has also shown the antagonistic effect of anti-CD47 on chimeric antigen receptor (CAR)-T cell therapy [112]. Antagonizing CD47 can also potentially disrupt immune homeostasis, leading to autoimmune reactions or exacerbated inflammatory conditions. Accordingly, blocking CD47 was shown to exacerbate inflammation and impair recovery in experimental autoimmune encephalomyelitis (EAE), an animal model of multiple sclerosis [113]. Studies have shown CD47’s role in angiogenesis and interaction with VEGFR or caveolin is crucial for vascular integrity. Hence, inhibiting CD47 can impair blood vessel function, affecting wound healing and tissue repair. Furthermore, CD47 inhibition may lead to unintended tissue damage or impaired regenerative capacity because of CD47’s role in survival and function of various cell types. Therefore, understanding the immune and non-immune functions of CD47 is crucial in developing strategies to mitigate adverse effects and for the safe and effective use of CD47-targeted therapies.

## Conclusions and future perspectives

**Fig. 5:**
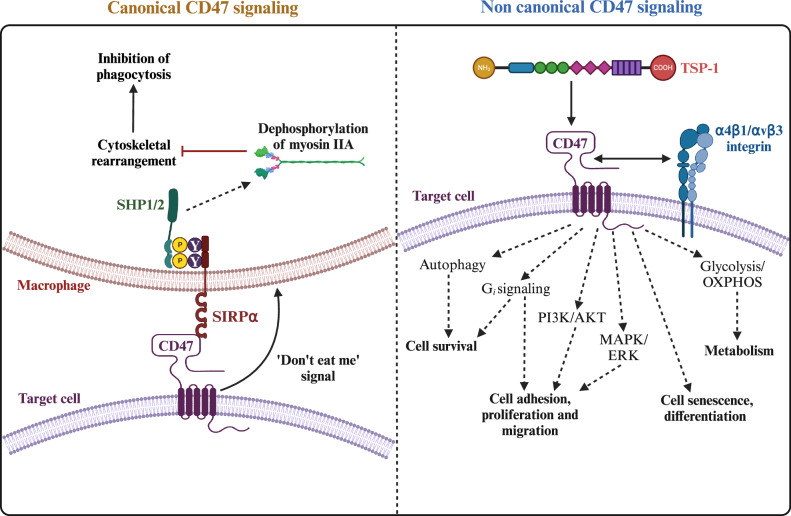
Overview of canonical versus noncanonical CD47 signaling. This figure was created using BioRender.com.

The role of CD47 extends beyond its classical function as a ‘don’t eat me’ signal in immune evasion. While it is well-established that CD47 prevents phagocytosis by interacting with SIRPα, recent research highlights its involvement in diverse cellular and metabolic processes through both SIRPα-dependent and -independent mechanisms. CD47 exhibits promiscuous binding to various ligands, including TSP1 and several integrins, indicating its involvement in mediating cell-specific functions beyond immune evasion (Fig. 5; Table 1). Moreover, the bidirectional nature of CD47-SIRPα signaling adds a further degree of complexity, which will warrant additional investigation.

**Table 1:** Cell-intrinsic functions of CD47 validated across different cell/tissue types.

CD47 function	Tissue/cell type
Inhibiting autophagic response under stress	Jurkat cells [36]
Inhibiting protective mechanisms against oxidative stress	Endothelial cells [20, 44], mouse lung tissue [35], Jurkat cells [38], vascular smooth muscle cells [22, 41, 44–46], mouse liver tissue [42], platelets [43]
Pro-apoptotic	Jurkat cells [23], B-CLL cells [50, 55, 56], T-ALL cells [51], breast cancer cells [52]
Anti-apoptotic	Cutaneous T lymphoma cells [58], thyroid carcinoma cells [59], thyroid cells [60]
Pro-adhesion/migratory/metastasis	RBCs [63], smooth muscle cells [64], intestinal epithelial cells [65, 74], ovarian cancer cells [67], colorectal cancer cells [71], Madin-Darby canine kidney (MDCK) cells [72], adamantinomatous craniopharyngioma cells [73], colorectal cancer cells [25], platelets [13], T cell lymphoma [27], neuroblastoma cells [77], non-small cell lung cancer cells [78], B-CLL [104]
Pro-proliferative	Colorectal cancer cells [25], adamantinomatous craniopharyngioma cells [73], astrocytoma/glioblastoma [79, 80], aortic smooth muscle cells [82], EBV-transformed B cells [83]
Promoting self-renewal	Hepatocellular carcinoma cells [90], breast cancer cells [91]
Inhibiting self-renewal	Intestinal epithelial cells [87], lung endothelial cells [88], renal tubular epithelial cells [89]
Maintaining senescence	Endothelial cells [93, 95], colorectal cancer cells [94]
Maintaining mitochondrial homeostasis	Jurkat cells [35], skeletal muscle cells [98], colorectal cancer cells [25], brown adipocytes [100]
Glycolysis	Colorectal cancer cells [25]
Inhibiting nucleotide biosynthesis	Jurkat cells [35], mouse lung tissue [38]
Fatty acid metabolism	Mouse liver tissue [101], brown adipose tissue [102]

Exploiting CD47 as a therapeutic target is challenged by potential off-target effects, which limit its clinical efficacy. Addressing these off-target effects while maintaining therapeutic efficacy requires a deeper understanding of the cell-intrinsic mechanisms and cell-type-specific functions of CD47. Overall, uncovering the noncanonical, cell-autonomous functions of CD47 is crucial for advancing our knowledge of its diverse roles in health and disease. This in-depth understanding will help pave the way for the development of therapeutic interventions that effectively target CD47-regulated pathways while mitigating potential off-target effects.
